# A Penny for Your Thoughts: Children’s Inner Speech and Its Neuro-Development

**DOI:** 10.3389/fpsyg.2019.01708

**Published:** 2019-08-14

**Authors:** Sharon Geva, Charles Fernyhough

**Affiliations:** ^1^Wellcome Centre for Human Neuroimaging, University College London, London, United Kingdom; ^2^Department of Psychology, Durham University, Durham, United Kingdom

**Keywords:** neural developmental mechanism, dorsal language pathway, ventral language pathway, arcuate fasciculus, superior longitudinal fasciculus

## Abstract

Inner speech emerges in early childhood, in parallel with the maturation of the dorsal language stream. To date, the developmental relations between these two processes have not been examined. We review evidence that the dorsal language stream has a role in supporting the psychological phenomenon of inner speech, before considering pediatric studies of the dorsal stream’s anatomical development and evidence for its emerging functional roles. We examine possible causal accounts of the relations between these two developmental processes and consider their implications for phylogenetic theories about the evolution of inner speech and the accounts of the ontogenetic relations between language and cognition.

## Development of Inner Speech

Inner speech – the experience of speaking silently in one’s head – is an enigmatic everyday phenomenon. It has been suggested to play an important role in psychological processes as diverse as memory, cognition, emotional regulation, auditory verbal hallucinations, and even consciousness and self-reflection (Alderson-Day and Fernyhough, 2015). Various domains of scholarship, including philosophy, psychology, and neuroscience, have seen renewed interest in inner speech, where it is seen as providing a context for exploring questions about the relationship between language and thought, the boundary between typical and atypical experience, and the emergence and maintenance of self-regulation (Fernyhough, 2016).

The origins of modern interest in inner speech can be traced to the Russian developmental psychologist, Vygotsky, who proposed that it develops through the gradual internalization of linguistic interactions that have been shaped by social interaction. Vygotsky argued that infants begin life embedded in social exchanges which, with the emergence of language, become linguistically mediated. In time, words that had previously been used to regulate the behavior of others are “turned back on the self” to regulate the child’s own behavior. In the preschool and early school years, such self-directed speech is mainly overt and audible, constituting a developmental stage known as private speech. With further development, these overt dialogues with the self become internalized so that they are entirely covert and inaudible, marking the development of inner speech.

Research in the last few decades has largely confirmed Vygotsky’s view of the development and functions of private and inner speech (Winsler et al., 2009). In particular, empirical studies have supported Vygotsky’s insight that private speech peaks in the preschool and early school years (between 4 and 7 years of age) and gradually reduces in frequency in middle childhood (Winsler et al., 2009). Although studying inner speech in childhood is fraught with difficulty, there is a consensus that this pattern corresponds to the emergence of fully internalized inner speech as private speech “goes underground” (Vygotsky, 1987), and the findings suggest that children begin to understand the concept of inner speech in the preschool and middle school years (Flavell et al., 1993, 1997, 2001; Fernyhough, 2009). Furthermore, there has been a growing recognition that overt self-directed speech (or private speech) continues to have important psychological functions into adulthood (Duncan and Tarulli, 2009). Fernyhough (2004) has proposed that adults can move flexibly between inner and overt private speech.

Studies of the various linguistic parameters in inner speech have so far focused on adult inner speech. Oppenheim and Dell (2008) have suggested that inner speech is phonetically impoverished in comparison to overt speech because inner speech lacks some of the phonetic components present in overt speech or because the internal monitoring system fails to detect the full range of phonetic features of the produced inner speech. However, others have shown that phonetics is fully specified in inner speech. For example, Corcoran (1966) has shown that readers automatically access phonetics in inner speech during silent reading. Özdemir et al. (2007) reported that the “uniqueness point”, the place in the sequence of the word’s phonemes at which it deviates from every other word in the language, influenced phoneme monitoring in inner speech suggesting that inner speech is specified to the same level as overt speech. Slevc and Ferreira (2006) documented a phonemic similarity effect in inner speech, again suggesting phonemic representation in inner speech. An fMRI study showed that manipulation of phonetic variables affects activation in phonological regions, even during a covert condition (Kell et al., 2017). Lastly, people’s ability to detect verbal transformations in inner speech (Sato et al., 2004) also suggests that the phonological representation is highly specified in inner speech. Others found that inner speech monitoring is influenced by lexical bias, suggesting that it is specified at the lexical level (Nooteboom, 2005; Geva and Warburton, 2019). While Slevc and Ferreira (2006) showed that monitoring of inner speech is not subject to the semantic similarity effect, this should not be simply interpreted as inner speech lacking semantic information. Rather, it might be that semantic information is not used for the task of monitoring errors. Lastly, recent studies have suggested that inner speech also carries prosodic information (Breen and Clifton, 2011; Filik and Barber, 2011; Geva and Warburton, 2019). However, it has been argued that information about prosody can be accessed by speakers even before inner speech is evoked (Coltheart et al., 1993; Rastle and Coltheart, 2000), and studies of tip-of-the-tongue somewhat support this argument (reviewed in Geva and Warburton, 2019).

Drawing on ideas of Vygotsky (1987), Fernyhough (2004) has suggested that inner speech can vary between fully specified expanded inner speech to a highly condensed form, with these variations reflecting levels of specification of syntax, semantics, and phonology. Expanded inner speech bears fully specified linguistic information and is similar to overt speech, while condensed inner speech lacks phonology (and all linguistic levels that follow, such as prosody and articulation) and full syntactic structure, and its semantics may be different to that of overt speech, such as being more idiosyncratic and personal in nature. Fernyhough (2004) further suggests that the transition from expanded to condensed inner speech is part of a developmental process and that adults can move flexibly between different forms of inner speech and overt private speech as conditions and task demands change.

## Neural Correlates of Inner Speech

With advances in neuroscientific methodology, attention has turned to the neural correlates of self-directed speech, although to date, this has mostly focused on inner speech in adults (Perrone-Bertolotti et al., 2014; Geva, 2018). Recent studies of inner speech function in adults with brain damage have shown that, for some patients, inner speech can be preserved while there is marked impairment in overt speech. More interestingly, other individuals can have preserved overt speech, but at the same time a salient impairment in inner speech (Geva et al., 2011a; Langland-Hassan et al., 2015; Stark et al., 2017). This dissociation suggests that somewhat distinct neural mechanisms support each type of speech. Although inner speech is (in the Vygotskian view) seen as developing out of overt speech, the process of internalization involves various types of semantic and syntactic transformation (Vygotsky, 1987) which make plausible the involvement of distinct neural substrates.

In the last 40 years, hundreds of functional imaging studies have examined the neural correlates of inner speech. These studies have used diverse tasks ranging from silent word repetition (Shuster and Lemieux, 2005; Pei et al., 2011), verb generation (Frings et al., 2006), stem completion (Rosen et al., 2000), and rhyme judgment (Paulesu et al., 1993; Pugh et al., 1996; Lurito et al., 2000; Poldrack et al., 2001; Owen et al., 2004; Hoeft et al., 2007) to silent reading (Bookheimer et al., 1995). Converging evidence from these studies of task-dependent inner speech points to the involvement of the left inferior frontal gyrus (IFG), and the left angular (AG) and supramarginal gyri (SMG) in the production and processing of inner speech (reviewed in Geva, 2018). These areas are connected *via* the dorsal language stream (Hickok and Poeppel, 2007; Saur et al., 2008), suggesting that it is involved in inner speech processing (Geva et al., 2011c; Rijntjes et al., 2012).

Spontaneous inner speech has only been scarcely studied, but findings so far support those from studies of task-dependent inner speech. A study by Doucet et al. (2012) found higher levels of spontaneous inner speech to be associated with increase in spontaneous fluctuations of activity (tested using resting state fMRI) in a fronto-parietal network, which includes the IFG, temporo-parietal junction, and superior temporal regions. In accordance with this result, it was shown that during resting state (while participants lie inside the scanner without performing any task and without exposure to any specific external stimulus), significant bursts of activation can be recorded in bilateral auditory cortex, which might be related to spontaneous occurrences of inner speech (Hunter et al., 2006). A detailed study of a single participant experiencing spontaneous inner speech in the scanner showed activation in left IFG and superior temporal sulcus (STS) as well as superior and middle temporal gyri during inner speech compared with rest. Left IFG activation was also present when comparing inner speech to other inner experiences (Kühn et al., 2014). In the only fMRI study that has directly compared spontaneous and elicited inner speech, a Region of Interest (ROI) analysis was used to contrast inner speech elicited by a task with occurrences of spontaneous inner speech. The results showed distinct patterns of activation associated with the two speech types, with left IFG activating in elicited, but not in spontaneous inner speech (Hurlburt et al., 2016). The implications of this finding are that it should not be assumed that activations associated with task-based inner speech reflect those found when inner speech arises spontaneously.

Buchsbaum and D’Esposito (2008) suggested that area Spt (Sylvian parietal temporal area, which is located within the Sylvian fissure at the parietal-temporal boundary), is the key area along the dorsal language stream that acts as an interface between the auditory-phonological system and the motor system. This function would implicate it in inner speech production in adults and would point to its potential as a starting point for exploring the neural substrates of inner speech in childhood. In the next sections, we present the current knowledge of dorsal stream anatomy and then discuss its development during childhood, as well as what is known about its function in pediatric populations.

## Dorsal Language Stream Anatomy

The dorsal language stream has been studied for more than a century, beginning with the seminal work of Dejerine (1895) and Wernicke (1874). It is specified in the classical Wernicke-Lichtheim-Geschwind anatomical model, where it is suggested that Broca’s area is connected to Wernicke’s area in the posterior temporal cortex *via* the arcuate fasciculus (AF). Advances in neuroimaging allowed further anatomical characterization of the dorsal language stream. In the past, connections between various areas in the human brain were mainly studied post-mortem. Today, the preferred methodology for defining anatomical white matter connections *in vivo* is diffusion tensor imaging (DTI). DTI images quantify the level and direction of the movement of water molecules in a tissue. As water molecules behave differently in different types of tissue, DTI can reliably distinguish between cell bodies (gray matter), tracts (white matter), and cerebrospinal fluid (CSF) (Pierpaoli et al., 1996; Pierpaoli and Basser, 1996; Basser and Pierpaoli, 1998). In recent years, DTI studies have refined, altered, and expanded upon the classical Wernicke-Lichtheim-Geschwind anatomical model of the language system (Hagoort, 2014). For terms related to DTI methodology, see Box – DTI Glossary. For a review of the use of DTI in language studies, see Geva et al. (2011b).

BOX **DTI Glossary (adapted from Geva et al., 2011b).**

*Diffusion Tensor Imaging* (DTI) – An MRI technique which is sensitive to the microscopic motion of water molecules in a tissue.

Diffusion tensor images are based on measurements of the movement of molecules:

*Isotropic movement* is a completely random movement which occurs in the absence of any restriction. This movement is equal in every direction and it is a characteristic of the movement of water molecules in neuronal cells (gray matter) and the cerebrospinal fluid (CSF).

*Anisotropic movement* is movement which occurs in the presence of physical restriction and is therefore larger in one direction. As axons restrict the movement of molecules parallel to the trajectory of the axon, the movement in the white matter is more anisotropic.

*Eigenvector* is the direction of movement of the water molecules (the diffusivity), while *eigenvalue* is the value of the diffusivity along the direction of the associated eigenvector. The *tensor* represents the overall movement of the water molecules, derived by averaging the strength of movement along the x, y, and z axes.

DTI studies commonly report the following parameters:

*Fractional Anisotropy (FA)* – A function of the eigenvalues, normalized to be between 0 (movement is completely unrestricted) and 1 (movement is restricted towards one direction), representing how similar the diffusivity values are in the different directions.

*Axial Diffusivity (AD)* – The value of the main (largest) eigenvalue. Also reffered to as *Longitudinal Diffusivity*.

*Radial Diffusivity (RD)* – The average of the two smaller eigenvalues. Also reffered to as *Transverse Diffusivity*.

*Mean Diffusivity (MD)* – The average of the three eigenvalues. This value describes the average distance traveled within a specific voxel.

*Apparent Diffusion Coefficient (ADC)* – The diffusion coefficient along a particular direction. In the context of DTI, MD and ADC are often used interchangeably.

**Figure 1:**
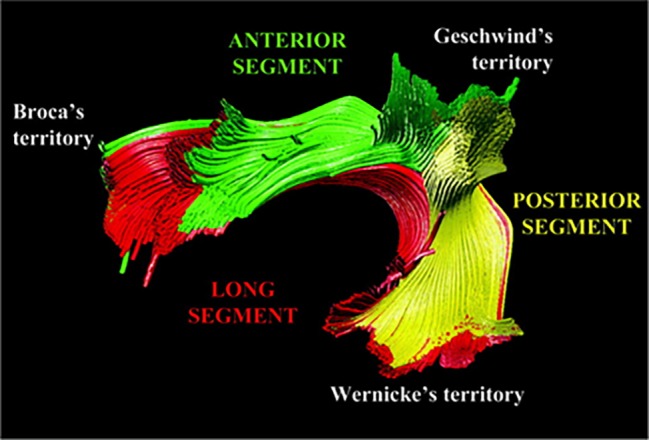
Tractography reconstruction of the three segments of the dorsal language pathway: the fronto-temporal (FT) segment/long segment (red); the fronto-parietal (FP) segment /anterior segment (green); and the temporo-parietal (TP) segment/posterior segment (yellow). The figure is adapted from Catani et al. (2005), and is being used with the permission of John Wiley and Sons.

Catani et al. (2005) suggested that in addition to the direct AF pathway between posterior temporal and inferior frontal regions (termed by Catani and colleagues *the long segment*), there are two other tracts: the anterior segment, which connects the posterior IFG with the inferior parietal lobe; and the posterior segment, which connects the inferior parietal lobe with the posterior temporal gyrus (see Figure 1). Later studies confirmed these findings in both adults (Parker et al., 2005; Frey et al., 2008) and children (Eluvathingal et al., 2007; Tak et al., 2016). These three segments are also referred to as the fronto-temporal (FT) segment (the long segment); fronto-parietal (FP) segment (the anterior segment), and temporo-parietal (TP) segment (the posterior segment) (Eluvathingal et al., 2007; see Table 1). In addition, imaging studies have suggested that a separate tract, the superior longitudinal fasciculus (SLF), also forms part of the dorsal language stream (Frey et al., 2008; Saur et al., 2008). The SLF can be divided into three components, of which only SLF III forms part of the dorsal language stream, connecting parietal area 40 (SMG), the ventral parts of peri-central Brodmann Areas (BA) 43, 2, 4, and 6, and BA 44 (pars opercularis) (Makris et al., 2005). SLF III differs from the long segment of the AF, which in its posterior part reaches the SMG (BA 40), posterior superior temporal gyrus (pSTG; BA 22), and the temporo-occipital region (BA 37). Lastly, it has been suggested that the dorsal pathway can be divided into two sections according to their frontal termination point: dorsal pathway I includes AF/SLF fibers which terminate at the premotor cortex, while dorsal pathway II includes AF/SLF fibers which terminate in the IFG BA 44 (Friederici, 2011, 2012). For details, see Table 1.

**Table 1:** Descriptions of the subcomponents of the dorsal language pathway, according to different studies.

Source	Fronto-temporal (FT) segment	Fronto-parietal (FP) segment	Temporo-parietal (TP) segment
Catani et al. (2005)	**Long segment of the AF/AF direct pathway**	**AF indirect anterior segment**	**AF indirect posterior segment**
	Runs medially and corresponds to classical descriptions of the arcuate fasciculus	Connects the IP cortex to Broca’s territory	Connects the IP cortex to Wernicke’s territory
Makris et al. (2005)	**Vertical AF**	**SLF III**	N/A
	Connects the caudal part of the STG, arches around the caudal end of the Sylvian fissure and extends to the lateral prefrontal cortex	Situated in the white matter of the parietal and frontal opercula; connecting the SMG, through Sylvian opercular white matter, to the ventral premotor cortex and IFG	
Martino et al. (2013)	**Long segment of the SLF/AF**	**Anterior segment of the perisylvian component of the SLF**	**Posterior segment of the perisylvian component of the SLF**
	Connects the ITG/MTG to the ventral pre-central gyrus	Connects the pSTG, just behind Heschl’s gyrus, and SMG, to the posterior portion of the frontal operculum: ventral pre-central gyrus (BA 6 and 4) and IFG	Connects pMTG to IP (AG)

*The description of “x to y” is arbitrary, as anatomical studies are blind to the directionality of the fibers. AF, arcuate fasciculus; AG, angular gyrus; BA, Brodmann area; IFG, inferior frontal gyrus; IP, inferior parietal; ITG, inferior temporal gyrus; MTG, middle temporal gyrus; pMTG, posterior middle temporal gyrus; pSTG, posterior superior temporal gyrus; SLF, superior longitudinal fasciculus; SMG, supramarginal gyrus; STG, superior temporal gyrus*.

Based on these anatomical definitions, the most likely tracts to support inner speech, within the dorsal language stream, are either the fronto-temporal or fronto-parietal segments. However, note that the exact anatomical end points of the various tracts are not agreed upon (see Martino et al., 2013 for an excellent discussion regarding the differences between various anatomical studies). In addition, in many imaging studies, these tracts are not distinguished, due to the methodological limitations of DTI, and are referred to as simply the dorsal stream or AF/SLF (Friederici, 2009).

In addition to the dorsal language stream, the human language system is supported by a ventral language stream (Hickok and Poeppel, 2007; Weiller et al., 2009), which mostly runs medially to the temporal lobe. This pathway connects occipital and temporal areas with frontal regions. It includes the inferior fronto-occipital fascicle (IFOF), which connects the occipital lobe, parietal lobe, and the posterior temporal cortex with the frontal lobe. In addition, the inferior longitudinal fascicle (ILF) connects the posterior occipito-temporal region and the temporal pole. Lastly, the uncinate fasciculus (UF) connects the anterior temporal cortex to inferior frontal areas (reviewed in Duffau, 2016).

## Pediatric Studies of the Dorsal Language Stream

### Anatomical Studies

The field of developmental cognitive neuroscience has seen a recent increase in interest in the role of the dorsal language stream in both typical development (Tak et al., 2016) and language and speech disorders (Morgan et al., 2016). In a pioneering study of its kind, full-term newborns were scanned within the first 3 days of life. DTI images showed that dorsal pathway I, which terminates in the premotor cortex, is already fully present at birth, while dorsal pathway II, which connects to the IFG, was undetectable (Perani et al., 2011). Similarly, in a study of language pathways among 6- to 22-week-old infants, it was shown that all language tracts were detectable at this age (both ventral and dorsal), although the AF showed the highest variability, terminating in the pre-central gyrus in most cases, and not reaching the IFG (Dubois et al., 2016). Among 0- to 54-month-olds, the SLF was found to be the least developed tract in the newborns, when compared to projection, callosal, brainstem, limbic, and other association fibers, and in fact, it could not be delineated before the age of 12 months (Hermoye et al., 2006). A study which included participants ranging the entire age span from neonates to adults showed that the SLF was difficult to identify in neonates and that it was significantly smaller in infants up to the age of 1 year. However, it could easily be identified in late childhood (6–10 years) (Zhang et al., 2007). While data from these studies converge to suggest that the dorsal language stream, or at least its portion which terminates in the IFG, is under-developed at birth, the explanation for this finding varies. Most authors interpret their findings as reflecting genuine anatomical difference between infants/children and adults (Hermoye et al., 2006; Zhang et al., 2007; Perani et al., 2011). However, Dubois et al. (2016) argue that the difference can be attributed to methodological issues, as studies of infants do not take into account the differences between the dorsal and ventral bundles in adults. Interestingly, post-mortem dissections of fetal human brains at 19–20 weeks gestational age showed that some of the ventral pathway, but not the dorsal one, is already present at this gestational age. In the ventral pathway, the external capsule (which contains the ILF and IFOF) was not clearly visible, but the UF was clearly identified. In healthy neonates, both the ILF and IFOF were identified, though they were not developed enough to reveal their projection to the frontal, temporal, and occipital lobes using DTI (Huang et al., 2006). The SLF was also not visible in the fetus, and it could also not be identified in the neonate. The temporal projection of the SLF was only clearly identifiable in the DTI scans of 5- to 6-year-olds (Huang et al., 2006). Hence, the finding of an existing ventral, but not dorsal, pathway in the fetus, suggests that the under-developed presentation of the dorsal pathway in DTI studies of infancy and childhood might be a genuine anatomical finding, rather than a methodological artifact. However, as the cause of death of the fetuses in the study by Huang et al. is not reported, and as fetal brains are rarely obtained without damage, these results should be interpreted with caution.

Further studies included school-aged children as well. Brauer et al. (2013) expanded on Perani et al.’s (2011) findings, showing that 7-year-olds already have both dorsal pathways I and II in place, similarly to adults, therefore obtaining very similar results to those obtained by Zhang et al. (2007). However, fractional anisotropy (FA) values, a commonly used DTI parameter (see Box – DTI Glossary), were still lower for 7-year-olds, compared to adults. Significant correlations between age and diffusivity parameters were found among cohorts of various age ranges [6- to 17-year-olds, examining the three segments of the AF (Eluvathingal et al., 2007); 4- to 17-year-olds, examining white matter integrity in the area of the AF (Paus et al., 1999); 5- to 18-year-olds, examining the AF (Schmithorst et al., 2002)]. Eluvathingal et al. (2007) distinguish between patterns of maturation based on different diffusivity parameters: The AF fronto-parietal segment showed a significant increase in FA with age, accompanied by significant decreases in mean, transverse, and axial diffusivity, suggesting increases in myelination. The authors suggest that this tract undergoes development mainly at the tested age range (6–17 years of age). The fronto-temporal and temporo-parietal segments of the AF showed significant age-related decreases in mean, transverse, and axial diffusivity measures that were not accompanied by significant increase in FA, which, according to the authors, suggest that much of the tracts’ maturation occurred before the age of 6 (Eluvathingal et al., 2007). A more recent DTI study of the maturation of the dorsal language pathway examined typically developing children in five age groups: 0–2, 3–5, 6–8, 9–11, and 12–14 years. It was found that the posterior segment developed first and actually showed an almost complete maturation already in the youngest age groups. This was followed by the anterior segment, which showed maturation in the middle age groups (around 6–8 years). Finally, the direct segment was suggested to mature only in the early teen years (Tak et al., 2016). Skeide et al. (2016) examined three age groups, similar to the middle ones of Tak et al. (2016), 3–4, 6–7 and 9–10-year-olds, as well as a group of adults. They showed a gradual and steady increase in FA of the dorsal pathway between the four age groups. While data in these studies suggest that the AF reaches maturation around the early school age years, non-linear relations were not statistically evaluated, and it is therefore difficult to determine at which age the maturation plateaus, signifying the age in which the language tracts reach an adult level of development. In addition, some of these studies did not include a group of adults, for comparing the level of maturation of the white matter tracts.

A few studies directly evaluated the age of maturation of various white matter tracts. Maturation was defined as the age at which diffusivity parameters reach a plateau. A longitudinal study which scanned children (aged 5–17) three times over a period of 3 years found increase in FA for both the AF and ILF, the latter forming part of the ventral language stream. However, the slopes were not dependent on initial age of testing, suggesting that the rate of change is equivalent across this age range (Yeatman et al., 2012). Studying participants aged 6–30 years Lebel et al. (2008) suggested that the AF reaches full maturation between the teen years and early 20s. A study of 7- to 68-year-olds found similar results, showing that all three segments of the dorsal language stream (anterior, posterior, and direct) reach full maturation around age 20–30 (Hasan et al., 2010). The authors further suggest that developmental studies should evaluate maturation of anatomical brain structures using non-linear relations.

**Figure 2:**
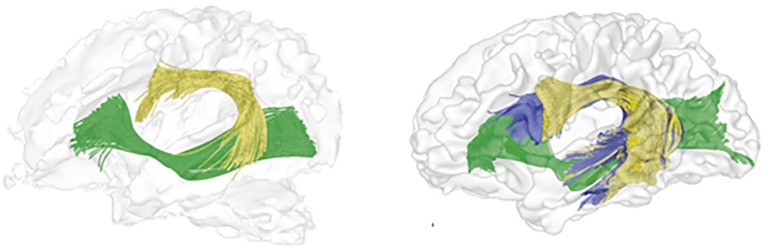
Tractography reconstruction of the left hemispheric language pathways, as they appear in the newborn (left) and adult (right) human brain. Tracts include: dorsal pathway I (AF/SLF fibers terminating at the premotor cortex) in yellow; dorsal pathway II (AF/SLF fibers terminating in the IFG BA 44) in blue; ventral pathway in green. The figure is adapted from Perani et al. (2011) and is being used with the permission of the authors and the National Academy of Sciences.

In summary, there is an agreement in the literature that the ventral language pathway is already detectable at birth (Perani et al., 2011; Tak et al., 2016) and matures faster than the dorsal language pathway (Brauer et al., 2013; Dubois et al., 2016; Tak et al., 2016). In addition, by late childhood, children’s dorsal pathway has similar anatomical structure to that of adults, although full maturation (as reflected in diffusivity parameters) is only achieved in the late teens or even early 20s (see Figure 2).

### The Functional Role of the Dorsal Pathway During Development

We have argued that the dorsal language stream supports the development and maintenance of inner speech. Much research has been done on the role of the dorsal language stream in language processing. Here, we ask whether some of the more well-established functions of this pathway have overlaps with inner speech and try to establish how it can support various and potentially distinct functions at the same time.

Two influential models of language development and processing assign specific functions to the dorsal language stream. The first describes language processing in general, suggesting that acoustic speech signals which are processed in posterior brain regions are transferred through the dorsal language stream to the frontal lobe, where they are converted into articulatory representations (Hickok and Poeppel, 2007). This process is essential for language acquisition, as infants and children learn to produce heard words (Hickok and Poeppel, 2007). Later in adulthood, this processing stream can be used for repetition (Saur et al., 2008; Kümmerer et al., 2013). However, based on the anatomical findings showing that the dorsal stream is under-developed in early childhood, developmental studies of the two language pathways suggested that, in early childhood, language development is actually dependent on the ventral pathway, not the dorsal one, while the dorsal pathway only subserves higher language functions which develop later (Brauer et al., 2013; Skeide et al., 2016).

Reconciling this apparent contradiction, Friederici (2009) suggested that language acquisition is dependent on dorsal pathway I, which terminates in the premotor cortex and develops early, while higher language functions depend on dorsal pathway II, which develops later and terminates more anteriorly in the IFG. This suggestion is supported by studies of adults learning an artificial language. In one study, a significant correlation was found between performance on an artificial language learning task and the integrity of the left long segment, which connects auditory and motor regions. No correlation was found between language learning and the integrity of any of the other language tracts examined (the anterior segment, the posterior segment, or the IFOF) (Lopez-Barroso et al., 2013). Another study demonstrated that performance on an artificial language learning task was reduced when participants’ subvocal rehearsal was blocked (using articulatory suppression), compared to a condition of no suppression, therefore allowing rehearsal. Additionally, task performance correlated with the integrity of the fibers running through the extreme capsule/external capsule, only when subvocal rehearsal was suppressed. The authors suggest that in adults, language learning without subvocal rehearsal is associated with the ventral pathway (Lopez-Barroso et al., 2011). Together, these studies suggest that the association between adult language learning and the dorsal pathway is mediated by inner speech, a suggestion that supports our hypothesis.

A second influential and extensively studied model describes the process of adult reading. According to the Dual-Route model (Paap et al., 1987; Paap and Noel, 1991; Coltheart et al., 1993; Rastle and Coltheart, 2000), word reading can be achieved through one of two routes. The first is a lexical route, dedicated to reading frequent regular, as well as irregular, words by means of whole word recognition. The second is the sublexical route, which supports the reading of new words and non-words, by utilizing direct grapheme to phoneme translation (but see connectionist models, for example Seidenberg and McClelland, 1989). It has been suggested that the lexical and sublexical routes are supported by the ventral and dorsal systems, respectively (Schlaggar and McCandliss, 2007). However, the dorsal portion relevant for reading was found to be the temporo-parietal segment (Pugh et al., 2000; Schlaggar and McCandliss, 2007; Vandermosten et al., 2012). Later studies have extended this model, adding the frontal segments (fronto-temporal and fronto-parietal) (Vanderauwera et al., 2017), showing their association with phonological awareness (reviewed in Vandermosten et al., 2012). Among a group of children aged 7–11, higher phonological awareness (the ability to parse the word into syllables and phonemes and manipulate these phonemes to make up new words) was associated with lower FA in the left AF, over and above age. The correlation was specific to the tract and task (compared with word reading, verbal short-term memory, and repetition tasks). The negative correlation is interpreted as experience-based successful pruning (Yeatman et al., 2011). A longitudinal study of 5-year-old pre-readers found similar results: children were tested at the start and end of their last nursery year, and it was found that better phonological awareness (end phoneme and rhyme identification tasks) was a significant predictor of FA in the left dorsal fronto-temporal segment, over and above naming and letter identification. This correlation was not found for the temporo-parietal segment (Vandermosten et al., 2015). Paralleling the early internalization of overt speech, studies have shown that during reading acquisition, children slowly switch from overt to covert reading (Kragler, 1995; Prior and Welling, 2001). However, studies have yet to test whether this transition is associated with anatomical developments of the ventral or the dorsal routes of language.

Studies of word learning and repetition emphasize a specific functional directionality of the dorsal language pathway, in which processing of input phonological data in posterior regions precedes retrieval of articulatory information in frontal regions, therefore suggesting that information propagates from posterior temporal to anterior frontal regions (Friederici, 2009; Agosta et al., 2010). Direct cortical stimulation of posterior language areas (SMG, middle and posterior STG and the adjacent middle temporal gyrus; MTG) of awake adults resulted in evoked potentials in anterior language areas (Broca’s area or adjacent regions), supporting the idea of processing progressing from posterior to anterior regions. However, in addition, stimulation of anterior regions also resulted in evoked potentials in all posterior regions tested (Matsumoto et al., 2004). A similar study using direct cortical stimulation in adult patients also showed bidirectional connectivity between pSTG and IFG (David et al., 2013), further suggesting that the connection is direct, and also providing evidence that propagation of information is faster from posterior to anterior regions, compared to the other direction. Koubeissi et al. (2012) also highlighted the bidirectionality of the connection, by showing that some patients have evoked response in posterior regions after stimulation of anterior regions, while others show the opposite response. Lastly, a neuro-computational model of the dorsal language stream also suggested a bidirectional transfer of information in this route (Schomers et al., 2017).

In summary, adult patient studies show that information propagates along both anterior and posterior directions within the human dorsal language pathway, and hence, one should be cautious in assuming posterior-to-anterior direction. Most developmental studies have so far focused on those language functions which are supported by unidirectional propagation of information in the dorsal route from posterior to anterior parts. We suggest that some reciprocal fibers in this pathway which send information in the other direction might be essential for inner speech development.

### The Dorsal Language Stream in Atypical Development

Some studies suggest a reduced use of inner speech among individuals with autistic spectrum disorder (ASD) (reviewed in Alderson-Day and Fernyhough, 2015). The reduction in inner speech use in some, but not all tasks, might be explained by the difference between dialogic and monologic thinking, with the former having its roots in communication with others, and the latter rooted in communication with the self (Fernyhough, 1996). Accordingly, it is expected that dialogic inner speech will be more affected among individuals with ASD (Alderson-Day and Fernyhough, 2015), a hypothesis that is confirmed in one study (Williams et al., 2012). A comprehensive review of DTI studies of ASD showed that people with ASD have white matter abnormalities across the brain, including in the AF/SLF, but not exclusively (Travers et al., 2012). In addition, correlations between diffusivity parameters and behavioral measurements have been inconsistent (Travers et al., 2012). A single study suggested that inner speech develops more slowly among children with specific language impairments (SLI), compared to typically developing children (Lidstone et al., 2012), but no neural correlates were studied. To the best of our knowledge, no other studies have examined inner speech in atypical pediatric populations. In cases where inner speech has been studied in atypical development, findings regarding white matter abnormalities are inconsistent, and associations with behavioral measurements vary greatly. However, this area of research offers an opportunity to further our understanding of the normal and abnormal development of inner speech and its neural correlates. We suggest that future studies of inner speech developmental abnormalities also examine whether behavioral performance correlates with dorsal stream anatomical integrity.

## Dorsal Language Stream – Inner Speech Hypothesis

By combining findings from different disciplines, we have presented evidence that the maturation of the dorsal language stream, especially the fronto-temporal and fronto-parietal segments, during childhood occurs in parallel with the development of inner speech. We therefore suggest that there is a link between these neuro-anatomical and psychological developments. This suggestion is based on findings from three separate lines of research. First, inner speech emerges around the early school years; second, the FT and FP segments of the AF/SLF mature around the same time; and third, adult studies suggest the involvement of those dorsal pathway segments in inner speech processing.

In addition, there is also more specific experimental evidence to support this hypothesis: firstly, studies suggested that language learning in adults is mediated by subvocal rehearsal and is correlated with the integrity of the dorsal tracts (Lopez-Barroso et al., 2011, 2013); and secondly, children’s performance on phonological awareness tasks, often requiring inner speech, is correlated with dorsal pathway development (Yeatman et al., 2011; Vandermosten et al., 2012, 2015).

Evidence for the parallel emergence of the neural pathway of the dorsal stream and the psychological process of inner speech should not, however, be interpreted uncritically as evidence for causation in any particular direction. The development of language is, of course, not solely influenced by maturation of brain structures. Large variability in both brain maturation and language abilities among individuals is partly due to environmental exposure (Kidd et al., 2018). It is well established that environment induces brain changes, especially during childhood (Sale, 2018). It is also known that induced white matter changes can be documented in animals *in vivo* (Sale, 2018) and in humans using DTI (Scholz et al., 2009). For example, in the area of language development, it has been shown that following 100 h of training program, poor readers showed changes in diffusivity parameters, suggesting increased myelination. Moreover, these changes occurred in the same frontal region where the children with poor reading ability showed lower FA than children with normal reading abilities. Lastly, changes were specific to the group which underwent the remediation program (Keller and Just, 2009). Together, these studies suggest that observed changes in brain maturation can be environmentally induced.

It would therefore be a mistake to assume that the emergence of inner speech is only developmentally constrained by dorsal pathway maturation. Following Vygotsky, Luria argued for bidirectional causation between biological maturation and sociocultural experience, fitting with the view that the internalization of social exchanges creates a new functional system of inner speech (Luria, 1965; Fernyhough, 2010). This view is in keeping with similar views of developmental interplay between interaction with the environment and biological maturation in the human brain (Gómez-Robles et al., 2015).

Lastly, we do not intend to minimize the role of the ventral language stream in inner speech development. Tasks requiring internal content analysis, as is the case in most occurrences of natural inner speech, probably rely on an interaction between the dorsal and the ventral streams (Rijntjes et al., 2012). However, as the ventral stream is already highly developed at birth, it is the maturation of the dorsal stream that presents the main constraint on inner speech development during childhood. Further research on the interplay between the ventral and the dorsal language streams may pay dividends for our understanding of functionally relevant distinctions between forms of inner speech, such as the distinction that can be made between subvocal rehearsal and planning (Alderson-Day and Fernyhough, 2015).

## A Comment on Inner Speech and the Origin of Language

Understanding the neurodevelopment of inner speech could be significant for current discussions about the origin of language in human evolution. There are contentious debates on whether language evolved as mechanism for symbolic thought (using inner speech) (Everaert et al., 2015, 2017) or as means of communication (Pinker and Jackendoff, 2005; Corballis, 2017). Jackendoff (1996) and others (Rijntjes et al., 2012) have discussed the importance of inner speech in human evolution, suggesting that the development of inner speech supported more complex and abstract thought. However, Pinker and Jackendoff (2005) emphasize that, in their view, language evolved initially as means of communication, and that inner speech is a “by-product”: a later evolutionary development which is a result of internalizing external speech, which in turn supports more complex thinking. Here, we extend this hypothesis to suggest that this evolutionary development is related specifically to anatomical changes in the dorsal language stream.

Comparative studies have found some substantial differences between dorsal stream tracts in humans, monkeys, and apes, suggesting an evolutionary change affecting these tracts. The human SLF III (the fronto-parietal segment) is similar to that of rhesus monkeys (Thiebaut de Schotten et al., 2012) and macaques (Croxson et al., 2005). The long segment of the AF, on the other hand, shows intra-species variations. In macaques (Rilling et al., 2008) and rhesus monkeys (Petrides and Pandya, 2009; Thiebaut de Schotten et al., 2012), AF connectivity in both anterior and posterior sites is limited. In these monkey species, the AF does not reach the middle or inferior temporal gyri in the posterior end and has less widespread connectivity in the anterior end. In chimpanzee, both parietal and frontal connectivities are wider than in the macaque; however, it is still not as developed as in humans (Rilling et al., 2008).

Additionally, in the macaque (Rilling et al., 2008) and rhesus monkey (Thiebaut de Schotten et al., 2012), the ventral pathway is substantially more developed than the dorsal pathway, as is the case in human infants (see section “Anatomical Studies”). The monkey ventral pathway resembles the human one in its anatomy (Croxson et al., 2005; Thiebaut de Schotten et al., 2012). In chimpanzees, the opposite is found: the dorsal pathway is more developed than the ventral one, as is the case in adult humans (Rilling et al., 2008).

Using neurocomputational modeling, Schomers et al. (2017) demonstrated that intra-species anatomical differences along the dorsal pathway are associated with functional differences. They suggest that compared with the monkey, the human anatomy of the dorsal pathway gives rise to stronger and longer-lasting neural activations, as well as parallel, rather than serial, activation (Schomers et al., 2017). They further suggest that the activity in the human model but not in the monkey model “can be viewed as reflecting (subvocal) articulation” (Schomers et al., 2017, p. 3051).

In summary, comparative studies show that monkeys and even chimpanzees have substantially less developed AF, compared with humans. It has already been suggested that changes in the dorsal tracts were the key element in human language evolution (Aboitiz and García, 2009; Friederici, 2009; Aboitiz, 2012). Aboitiz and colleagues further argue that these changes gave rise to inner speech and its associated function: phonological working memory (Aboitiz and García, 2009; Aboitiz, 2012). If early humans had under-developed AF, and if highly developed AF is the neural substrate for inner speech production (as we argue here), then, one might suggest that early humans had no, or at least limited, inner speech. In the absence of inner speech, language would have been initially used as means of communication (Pinker and Jackendoff, 2005; Corballis, 2017) rather than as mechanism for symbolic thought (Everaert et al., 2015, 2017).

Another line of evidence connecting inner speech with language evolution comes from genetic studies. The FOXP2 gene has long been associated with speech and language in humans (Lai et al., 2001; Vargha-Khadem et al., 2005), and later, it has been argued that both FOXP2 and its target genes have undergone adaptive protein evolution during human evolution (Enard et al., 2002; Zhang et al., 2002). The FOXP2 gene was first identified in the KE family, whose affected members have a mutation in this gene, and they suffer from speech and language deficits (Lai et al., 2001). A later study has shown that those affected individuals suffer from phonological loop impairments, even when the task requires only inner speech, with no overt recitation (Schulze et al., 2018). Others have shown an association between FOXP2 mutations and auditory hallucinations in schizophrenia (Sanjuan et al., 2006; Tolosa et al., 2010). Building on these findings, Crespi et al. (2017) have studied more than 800 healthy individuals, finding an association between a specific variant of the gene and inner speech scores (based on self-rating). Together, these studies link inner speech to one of the main genes implicated in the evolution of language, putting inner speech as a main component in the evolution of language as a whole (Crespi et al., 2017).

Lastly, we do not argue that the ontogeny (of inner speech) recapitulates its phylogeny. That is, the anatomical changes in the language pathways that occur during embryonic development and early childhood are somewhat different from those that came about in the course of evolution. The bidirectional causal view that we have espoused here is in keeping with the finding that human infants are born without a fully matured dorsal pathway. It is the development of this neural system, in parallel with human infants’ socially and linguistically patterned experience, that makes the emergence of inner speech possible.

## Conclusion

The anatomy of the arcuate fasciculus was described more than 200 years ago, and its role in language processing has been discussed extensively (Catani and Mesulam, 2008). Together with subcomponents of the SLF, it forms the dorsal language stream. Neurodevelopmental studies have shown that humans are born with a dorsal language stream which is not fully developed and that it slowly matures throughout early childhood. Based on the temporal co-occurrence of dorsal stream maturation and the emergence of inner speech in children, as well as findings from studies of language development and adult language processing, we have suggested that the maturation of the dorsal language stream is closely linked to inner speech development. Studies of the neural mechanisms associated with inner speech in children are scarce. However, recent methodological advances in the study of neuro-development (Satterthwaite et al., 2014) and brain networks (Bassett and Bullmore, 2017) on the one hand, and inner speech (Geva and Warburton, 2019) on the other hand, can all contribute to our ability to make progress in this area. By linking findings from different disciplines, studies on the neural mechanisms of inner speech development can further our understanding of the role of inner speech and bridge the gap between research into language, cognition, development, and evolution.

## Author Contributions

SG initiated the article and conducted the literature review. Both authors drafted parts of the manuscript and critically revised the work for intellectual content. Both authors approved the submitted version.

### Conflict of Interest Statement

The authors declare that the research was conducted in the absence of any commercial or financial relationships that could be construed as a potential conflict of interest.
